# Supplementary material for: Carbonyl Cyanide 3-Chloro Phenyl Hydrazone (CCCP) Restores the Colistin Sensitivity in Brucella intermedia
Source: Int J Mol Sci. 2023 Jan 20;24(3):2106. doi: 10.3390/ijms24032106 (PMC9917161; doi:10.3390/ijms24032106)

International Journal of Molecular Sciences

Supplementary materials

Carbonyl Cyanide 3-Chloro-Phenyl hydrazone (CCCP) restores the colistin sensitivity in *Brucella intermedia*

Malak Zoaiter[1,2], Zaher Zeaiter[4] , Oleg Mediannikov[1,3], Cheikh Sokhna[1,3,5], Pierre-Edouard Fournier[1,2]*

[1] IHU-Méditerranée-Infection, Marseille, France

[2] Aix-Marseille Univ, IRD, AP-HM, SSA, VITROME, Marseille, France

[3]Aix-Marseille Univ, IRD, AP-HM, MEPHI, Marseille, France

[4] Department of Biology, Faculty of Sciences, Lebanese University LU, Beirut, Lebanon

[5] Campus Commun UCAD-IRD of Hann, Dakar, Senegal

[*]**Corresponding author:** Pierre-Edouard Fournier

Aix-Marseille Université, Institut Hospitalo-Universitaire Méditerranée Infection,

19-21 Boulevard Jean Moulin 13385 Marseille cedex 05, France.

Phone: +33(0)413732401. Fax: +33(0)413732402

E-mail: pierre-edouard.fournier@univ-amu.fr

Figure S1 Observation of the effect of CCCP alone and in combination with CO using the liquid method **(a)** and the agar method **(b)** on Q1107, Q1109 and Q1111 *Brucella* strains. **(a):** CO-MIC determination by the microdilution method with and without CCCP 10 µg/ml . **(b):** left two columns, CO inhibition diameter on CAMH plates containing 0.2% DMSO or CCCP 10 µg/ml ; right, growth of strains on CAMH + CCCP 10 µg/ml without CO.The 2 photographs were not to scale.

CO: Colistin; **CCCP:** Carbonyl Cyanide 3-Chloro Phenyl hydrazone; **MIC:** Minimal Inhibitory Concentration; **DMSO:** Di-Methyl-Sulfoxide; **CAMB**: Cation Adjusted Muller Hinton; **Ctrl:** Control bacterial growth

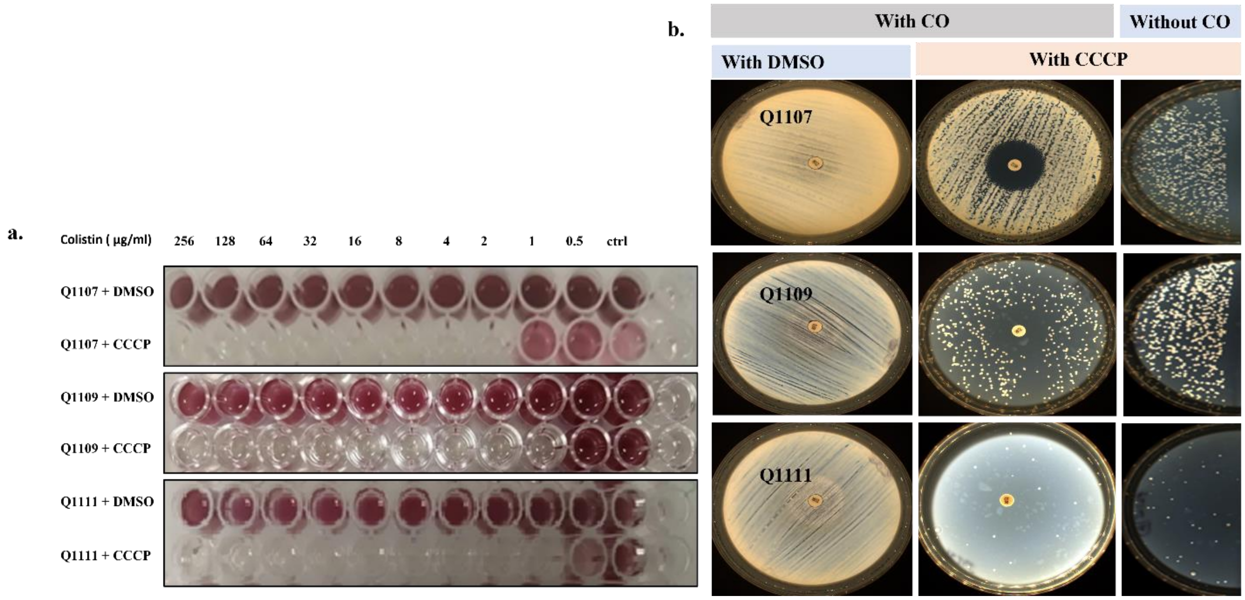

Figure S2 Growth curves of *B. intermedia* strain Q1103 in different CO concentrations with 50 µg/ml of the following efflux pump inhibitors **(a)** PaβN (P<0.022 **(b)** RSP (P<0.0002) and **(c)** VRP (P<0.0011).

CO: Colistin; **CCCP:** Carbonyl Cyanide 3-Chloro Phenyl hydrazone; **RSP:** Reserpine; **VRP:** Verapamil; **PaβN:** phe-arg β-naphtylamide dihydrochloride

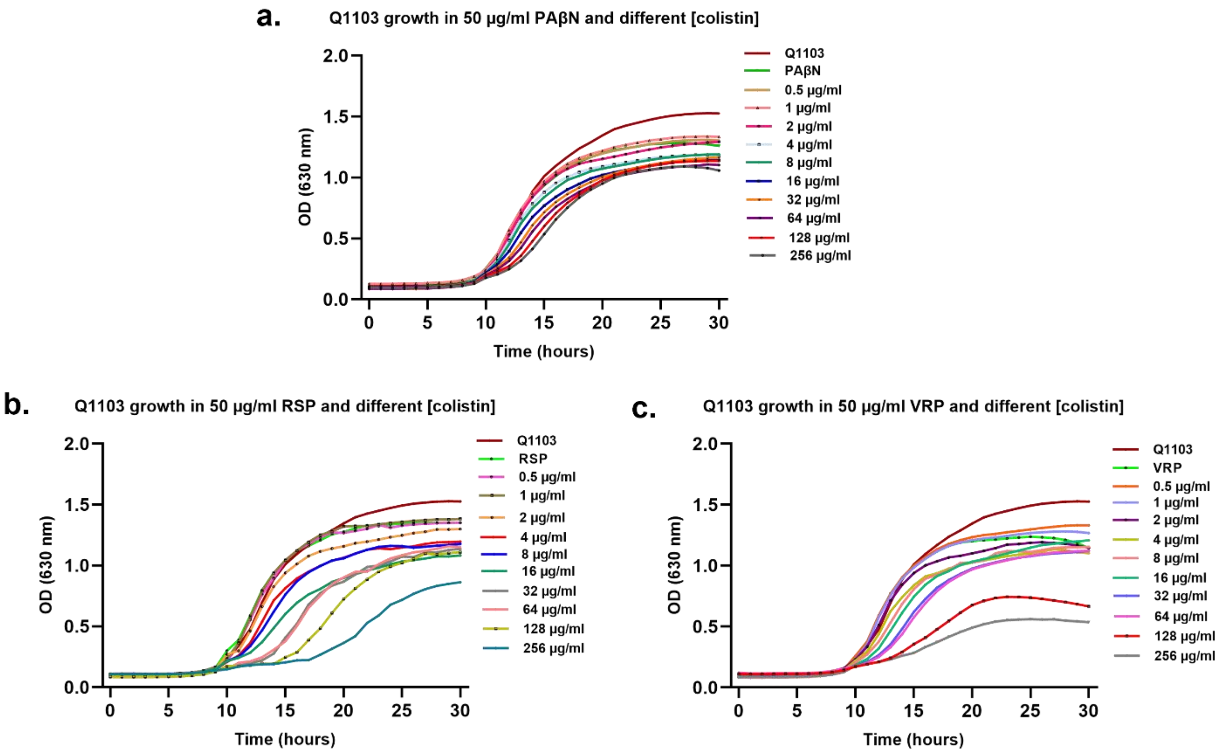

Supplement: Supplementary file 1 [file ijms-24-02106-s001.zip › ijms-2132892-supplementary.pdf]
